# The Effect of Renal Denervation on Capillary Density in Patients With Uncontrolled Hypertension

**DOI:** 10.1111/micc.70015

**Published:** 2025-06-22

**Authors:** Lefki Nikolopoulou, Kyriakos Dimitriadis, Nikolaos Pyrpyris, Fotios Tatakis, Panagiotis Iliakis, Costas Thomopoulos, Dimitrios Konstantinidis, Loukianos Rallidis, Dimitrios Tousoulis, Konstantinos Tsioufis

**Affiliations:** ^1^ First Department of Cardiology, School of Medicine National and Kapodistrian University of Athens, Hippokration General Hospital Athens Greece; ^2^ Department of Cardiology General Hospital of Athens “Laiko” Athens Greece; ^3^ Second Department of Cardiology, School of Medicine National and Kapodistrian University of Athens, University General Hospital ATTIKON Athens Greece

**Keywords:** albuminuria, capillary density, capillary rarefaction, hypertension, microcirculation

## Abstract

**Objective:**

Hypertension is related to the pathogenesis of microvascular dysfunction. Renal denervation is a guideline‐endorsed intervention for the management of uncontrolled hypertension. However, the effect of renal denervation on skin capillary density, as assessed by nailfold capillaroscopy, is unknown.

**Methods:**

Individuals with stage I/II uncontrolled hypertensions were enrolled and allocated to either undergo renal denervation or serve as controls. Nailfold capillaroscopy was performed at baseline and at 12 months. Furthermore, the albumin to creatinine ratio (ACR) and office/ambulatory blood pressure (BP) levels were monitored throughout the study.

**Results:**

A total of 45 individuals (28 renal denervation, 17 control) were enrolled in our study. No difference was found in baseline capillary density. At 12 months, all patients had controlled BP, while the denervation arm had a significantly greater number of capillaries, compared with control (90.9 ± 14.0 vs. 82.5 ± 10.6 capillaries/mm^2^; *p* = 0.036). However, the change from baseline capillary density was not significantly different between groups (4.6 ± 6.1 vs. 1.39 ± 8.8 capillaries/mm^2^; *p* = 0.150). Moreover, the change of ACR was not different between groups (−2.7 ± 13.8 vs. 0.46 ± 5.2; *p* = 0.365).

**Conclusion:**

In patients with uncontrolled stage I/II hypertension, renal denervation may have a beneficial effect on skin capillary density.

## 1 Introduction

Essential hypertension is one of the most prevalent cardiovascular pathologies in the globe. Recent data from the Global Disease Burden Analysis indicate a worldwide prevalence of approximately 33.1%, with higher rates observed in the European region (36.9%) [1]. The effects of hypertension in cardiovascular (CV) and overall health are well recognized, having established associations with all‐cause and cardiovascular mortality [2]. However, hypertension control in the recent years has not improved [3], while hypertension‐related mortality in 2021, despite having a downward trend, still remains at an estimated annual death rate of 138 deaths per 100 000 people [1]. More recently, the link of hypertension with microcirculation has been more extensively examined. Studies have shown that, at the microvascular level of patients with hypertension, there is an adverse, eutrophic remodeling of resistance arteries and rarefaction of capillary arteries observed, further increasing peripheral vascular resistance [4]. Such changes are regulated by a handful of mechanisms, including genetics, sympathetic nervous system (SNS) overactivation, vascular inflammation and endothelial dysfunction [5]. Pathophysiologically, capillary rarefaction can be functional, resulting from reduced NO bioavailability or increased levels of endogenous vasoconstrictors, structural, due to chronic vasoconstriction and hypoperfusion or reduced vascular growth factors, or mixed [6]. Such alterations have been found to be reversed with antihypertensive treatment [7].

The arsenal of hypertension management has been expanded, in the recent years, with the addition of invasive procedures modulating SNS overactivation. The most studied is renal denervation (RDN), aiming to ablate the renal sympathetic nerves, which are in close proximity to the renal artery lumen, thus reducing sympathetic overactivation and achieving hypertension control [8]. The effects of RDN have been documented in numerous randomized clinical trials, with sustained, long‐term blood pressure (BP) reductions [9], resulting in the recommendation of RDN in the 2023 European Society of Hypertension (ESH) guidelines on the Management of Arterial Hypertension, for patients with uncontrolled hypertension [10]. Given the effect of antihypertensive treatment in microcirculation and capillary density, similar results could be also reported with RDN. Thus, the aim of this study was to examine the effect of RDN on microcirculation via capillary density in patients with uncontrolled stage I/II hypertension.

## 2 Methods

### 2.1 Study Population

The study population of this study consisted of 45 consecutive adult patients with uncontrolled Grade I or Grade II hypertension, with or without medication, that were referred to our outpatient hypertension unit. In specific, the patients were suitable for inclusion if they had an office systolic BP of 140–169 and/or a diastolic BP of 90–109, and/or a 24‐h mean systolic and diastolic BP greater than 130 and/or 80, respectively, as defined in the recent ESH guidelines [10]. Exclusion criteria included secondary hypertension, diabetes mellitus, known cardiovascular disease, severe sleep apnea syndrome, body mass index (ΒΜΙ) > 40 mg/m^2^, renal artery disease or previous renal artery interventions, known chronic kidney disease (CKD), defined as estimated glomerular filtration rate (eGFR) < 60 mL/min per 1.73 m^2^ or critical illness. Patients meeting the inclusion criteria were assigned (3:2) to either undergo RDN or serve as controls.

The study protocol complies with the Declaration of Helsinki and was approved by our institutional ethics committee. Additionally, all participants gave written informed consent.

### 2.2 Protocol and Procedures

#### 2.2.1 Anthropometric Characteristics and Laboratory Measurements

All studies were carried out during the morning. At the baseline visit, demographic and anthropometric data and laboratory tests were collected from all patients. Patients' BMI was measured according to the World Health Organization Guidelines, that is by dividing each participant's body weight by the squared height. Moreover, eGFR was calculated using the MDRD equation. Patients also underwent standard transthoracic echocardiographic examination. These measurements were repeated at 3‐, 6‐, and 12‐month follow‐ups.

#### 2.2.2 Blood Pressure Measurement

The measurement of BP was performed according to European guidelines [10, 11]. All measurements were made with an accredited office BP device (Omron 705CP‐II automatic BP monitor). Three measurements were taken in each patient, with the BP value being the mean of the second and third observations. Regarding ambulatory blood pressure measurement, an accredited device was used (Mobil‐O‐Graph; I.E.M GmbH, Stolberg). The device was measuring BP every 20 min throughout the examination period, in order to have homogeneous results. The 24 h mean BP will be the mean of BP measurements recorded throughout the day, as well as daytime and nighttime for the evaluation of morning and night BP levels. BP was measured at the 3‐, 6‐, and 12‐month timepoints. Additional treatment decisions were made based on the BP values of each visit, in order for patients to achieve optimal BP control according to guidelines [10].

#### 2.2.3 RDN

Patients assigned to the RDN cohort underwent a baseline renal angiogram, in order to confirm anatomic suitability for the procedure. The intervention was performed under local anesthesia, via the femoral access, in the catheterization laboratory of our department. The Spyral system (Medtronic, Minneapolis, MN, USA) was used for all procedures. Ablation was performed to all suitable renal artery branches (proximally and distally), in order to ensure a successful result. All patients stayed overnight in the hospital for observation and monitoring of potential procedure‐related complications.

#### 2.2.4 Estimation of Microalbuminuria

The extent of albuminuria was recorded in all patients at baseline and at the discrete follow‐up time points. An early morning spot urine sample was acquired, and albuminuria was assessed by the quantitative detection of albumin concentration (A), creatinine concentration (C), and albumin to creatinine ratio (ACR) via a specific analyzed (Bayer DCA 2000+). Microalbuminuria was evaluated at baseline and at the 12‐month follow‐up.

#### 2.2.5 Nailfold Capillaroscopy

All patients underwent nailfold videocapillaroscopy using a high‐definition digital videomicroscope (VideoCap 3.0; DS Medica, Italy) on the dorsum of the nails of the upper extremities. Before the examination, the patient's hand was placed at the heart levels and a drop of vegetable oil was used in each of the nailfold that was going to be examined. The videocapillaroscope directly contacted each patient's nailfold, with appropriate alterations of the contact angle and direction in order to obtain the best achievable image quality. Four consecutive images (1 × 1 mm in size) were taken at 200 times magnification from the middle of the examined nailfold. The examination was performed at baseline and at 12 months post‐RDN. Capillary density (CD) was defined as the number of capillaries in a one‐millimeter span of the distal row of the finger.

### 2.3 Statistical Analysis

Quantitative variables were expressed as mean (standard deviation) or as median (interquartile range). Qualitative variables were expressed as absolute and relative frequencies. For the comparison of proportions chi‐squared and Fisher's exact tests were used. Students' *t*‐tests and Mann–Whitney tests were used for the comparison of continuous variables between two groups. Repeated measurements analysis of variance (ANOVA) was adopted to evaluate the changes observed in all under study parameters between the two study groups over the follow‐up period. Log transformations were made in case of not normal distribution. Statistical significance was set at *p* < 0.05, and analyses were conducted using SPSS statistical software (version 22.0).

## 3 Results

### 3.1 Baseline Characteristics

A total of 45 patients were included in this study, out of whom 27 (60%) underwent RDN, and 18 (40%) served as controls. Baseline characteristics are presented in Table 1. The mean age of the entire cohort was 49.9 ± 8.7 years, with 22.4% of patients being female. Included patients had similar rates of comorbidities, as well as body weight and renal function. No significant change was found in the BMI (ΔBMI: −0.3 ± 1.7 vs. −0.2 ± 2.8 kg/m^2^; *p*: 0.902) and eGFR values (eGFR: −9.6 ± 17.3 vs. −8.3 ± 13.2 mL/min/1.73m^2^; *p* = 0.792) of the two cohorts between baseline and 12‐month follow‐up.

**TABLE 1:** Baseline characteristics.

	RDN (*n* = 27)	Control (*N* = 18)	*p*
Gender (male, %)	23 (85.1)	12 (66.7)	0.167
Age	48.4 ± 8.1	52.1 ± 8.1	0.171
Smoking	10 (37.0)	7 (38.9)	—
Packs/year	11.9 ± 12.3	20.6 ± 43.0	0.324
Hypertension diagnosis (years)	3.5 ± 5.1	3.0 ± 4.7	0.324
Dyslipidemia (%)	7 (25.9)	7 (38.9)	—
OSAS (%)	2 (7.4)	2 (11.1)	—
BMI (kg/m^2^)	30.6 ± 5.5	30.4 ± 4.8	0.911
eGFR (mL/min/1.73 m^2^)	133.3 ± 35.6	118.3 ± 24.0	0.126

### 3.2 Blood Pressure

Baseline office and ambulatory BP measurements are shown in Table S1. Patients in the RDN group had significantly greater office systolic (164.3 ± 8.4 vs. 152.0 ± 10.6 mmHg; *p* < 0.001) and diastolic BP (104.5 ± 7.7 vs. 96.3 ± 8.0 mmHg; *p* = 0.001), as well as mean 24‐h systolic (149.7 ± 4.6 vs. 144.6 ± 7.8 mmHg; *p* = 0.008) and diastolic BP (101.6 ± 13.1 vs. 92.6 ± 9.0 mmHg; *p* = 0.014). Regarding BP levels during the day, individuals enrolled in the renal cohort also had significantly elevated systolic (143.9 ± 8.0 vs. 135.2 ± 11.0 mmHg; *p* = 0.004) and diastolic nighttime BP (94.2 ± 9.7 vs. 84.6 ± 9.2 mmHg; *p* = 0.002). Baseline heart rate (HR) did not differ between groups in both baseline office and 24‐h ambulatory measurements.

**FIGURE 1:**
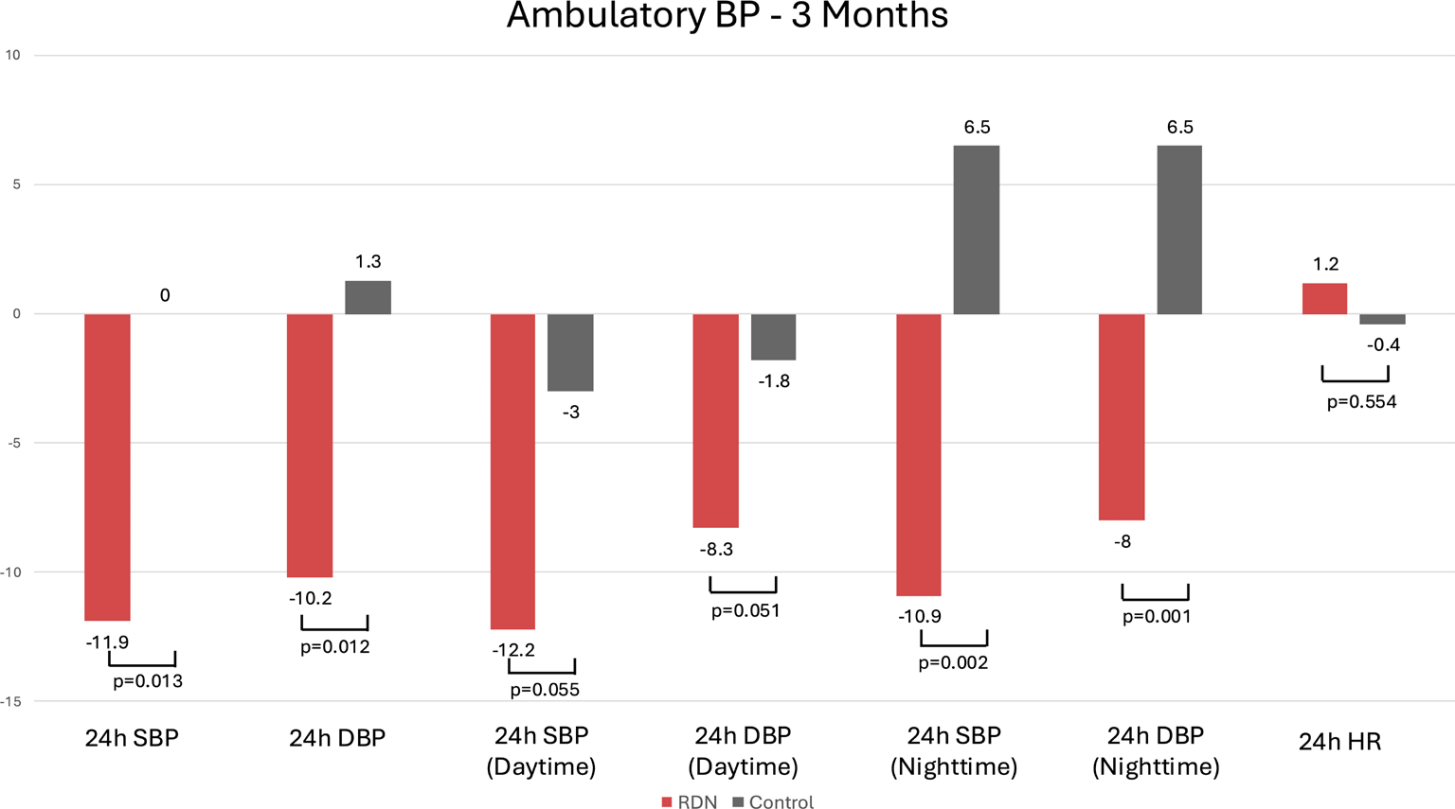
Changes in ambulatory BP levels from baseline at 3 months. BP, blood pressure; DBP, diastolic blood pressure; HR, heart rate; RDN, renal denervation; SBP, systolic blood pressure.

Following RDN, BP levels were evaluated at 3‐, 6‐, and 12‐month follow‐ups. The absolute values of BP did not differ significantly between the RDN and control group throughout the follow‐up, although some non‐significant trend toward improvement was shown in the 3‐month 24‐h mean systolic BP and 24‐h nighttime systolic BP (*p* = 0.054 and *p* = 0.055, respectively) (Table S2). However, when calculating the treatment difference (change in BP from baseline, ΔBP) between the two arms, there were significant differences in the reduction of BP at 3 months in favor of RDN, in both 24 h mean systolic and diastolic BP, as well as 24 h nighttime systolic and diastolic BP (Table 2, Figure 1). A non‐significant trend toward increased BP reduction in the RDN group was noted in 24 h daytime systolic and diastolic BP (*p* = 0.055 and *p* = 0.051, respectively). At 6‐ and 12‐month follow‐ups, in most BP measurements, no significant difference was found from baseline between groups (Table 2, Figure S1).

**TABLE 2:** BP reductions at 3‐, 6‐ and 12‐month follow‐ups.

	Renal denervation (*n* = 27)	Control (*n* = 18)	*p*
24 h‐SBP (mmHg)			
3 months	−11.9 ± 11.5	0.00 ± 10.3	**0.013**
6 months	−17.3 ± 13.0	−14.5 ± 10.9	0.513
12 months	−20.4 ± 9.9	−16.9 ± 8.2	0.217
24 h‐DBP (mmHg)			
3 months	−10.2 ± 11.6	1.3 ± 5.6	**0.012**
6 months	−15.6 ± 12.0	−9.38 ± 7.8	0.100
12 months	−17.4 ± 11.7	−11.0 ± 8.7	0.055
24 h‐SBP (daytime) (mmHg)			
3 months	−12.2 ± 12.0	−3.0 ± 9.3	0.055
6 months	−16.9 ± 13.1	−15.9 ± 11.8	0.804
12 months	−21.4 ± 10.6	−18.2 ± 8.7	0.296
24 h‐DBP (daytime) (mmHg)			
3 months	−8.3 ± 8.7	−1.8 ± 5.0	0.051
6 months	−13.3 ± 8.4	−10.2 ± 8.9	0.298
12 months	−15.8 ± 8.6	−12.3 ± 9.7	0.218
24 h‐SBP (nighttime) (mmHg)			
3 months	−10.9 ± 12.7	6.5 ± 13.2	**0.002**
6 months	−17.6 ± 15.0	−12.5 ± 11.3	0.297
12 months	−18.6 ± 11.5	−15.8 ± 14.0	0.468
24 h‐ DBP (nighttime) (mmHg)			
3 months	−8.0 ± 9.7	6.5 ± 8.9	**0.001**
6 months	−14.3 ± 10.5	−8.5 ± 7.1	0.087
12 months	−15.0 ± 8.3	−10.1 ± 8.9	0.068
24 h‐HR (beats/min)			
3 months	1.2 ± 6.9	−0.4 ± 3.6	0.554
6 months	−0.7 ± 7.2	−2.8 ± 3.9	0.349
12 months	0.59 ± 7.6	−0.61 ± 9.2	0.994

*Note:* Statistical significance *p*‐value < 0.05.

Abbreviations: DBP, diastolic blood pressure; HR, heart rate; SBP, systolic blood pressure.

Importantly, in the RDN group, there were no major short‐ or long‐term procedure‐related adverse effects, including significant renal artery stenosis, renal artery rupture, major bleeding, significant renal function deterioration, throughout the 1‐year follow‐up.

### 3.3 Capillary Density

**FIGURE 2:**
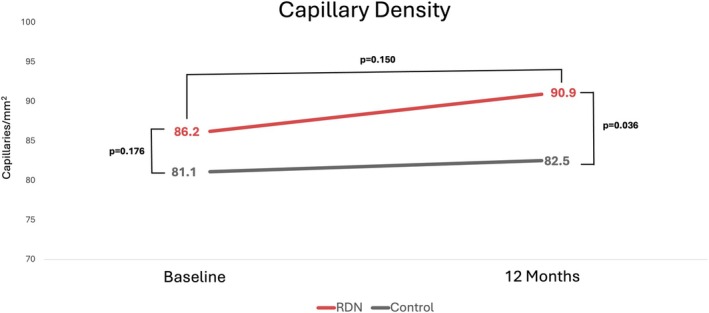
Changes in capillary density from baseline to 12‐month follow‐up. RDN, renal denervation.

Nailfold capillaroscopy showed comparable capillary density between patients at baseline (86.2 ± 2.9 vs. 81.1 ± 11.0 capillaries/mm^2^; *p* = 0.176). At 12‐month follow‐up, capillary density was significantly different, with greater number of capillaries noted in the RDN group (90.9 ± 14.0 vs. 82.5 ± 10.6 capillaries/mm^2^; *p* = 0.036). The change in capillary density from baseline, despite being numerically higher in the RDN arm, did not reach statistical significance (4.6 ± 6.1 vs. 1.39 ± 8.8 capillaries/mm^2^; *p* = 0.150) (Table 3, Figure 2).

**TABLE 3:** Changes in capillary density.

	Renal denervation (*n* = 27)	Control (*n* = 18)	*p*
Capillary density, baseline (capillaries/mm^2^)	86.2 ± 12.9	81.1 ± 11.0	0.176
Capillary density, 12 months (capillaries/mm^2^)	90.9 ± 14.0	82.5 ± 10.6	**0.036**
Change from baseline (capillaries/mm^2^)	4.6 ± 6.1	1.39 ± 8.8	0.150

*Note:* Statistical significance *p*‐value < 0.05.

### 3.4 Microalbuminuria

**FIGURE 3:**
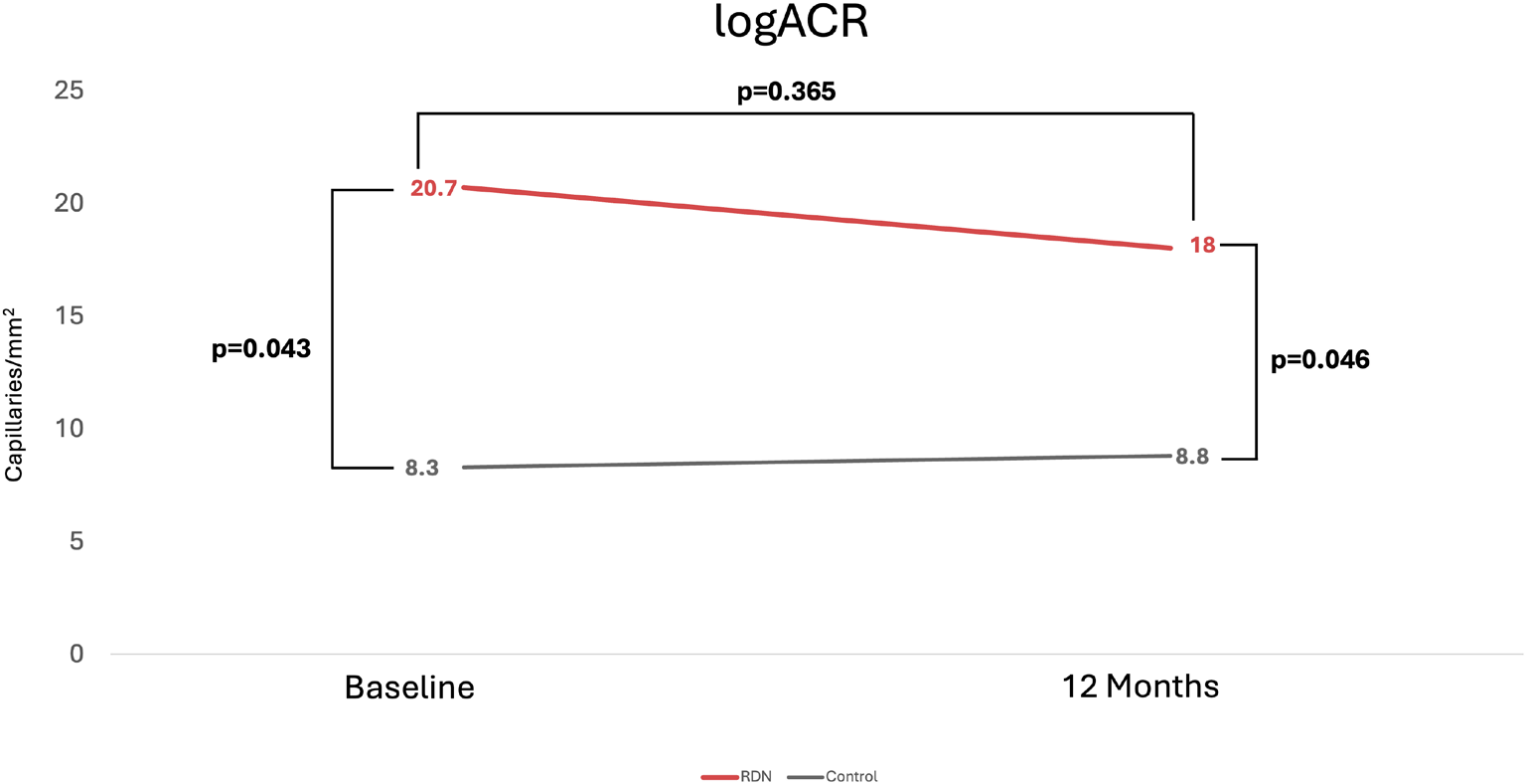
Changes in albumin/creatinine ratio from baseline to 12 month follow‐up. RDN, renal denervation.

At baseline, ACR was significantly higher in patients undergoing RDN (20.7 ± 33.8 vs. 8.3 ± 4.2 mg/g; *p* = 0.043), as were at 12‐month follow‐up, despite a decrease in the RDN group (18.0 ± 20.8 vs. 8.8 ± 4.8 mg/g; *p* = 0.046). No difference was found in the change of ACR from baseline at 1 year (−2.7 ± 13.8 vs. 0.46 ± 5.2 mg/g; *p* = 0.365) (Table 4) (Figure 3).

**TABLE 4:** Changes in albumin/creatinine ratio.

	Renal denervation (*n* = 27)	Control (*n* = 18)	*p*
logACR, baseline (mg/g)	20.7 ± 33.8	8.3 ± 4.2	**0.043**
logACR, 12 months (mg/g)	18.0 ± 20.8	8.8 ± 4.8	**0.046**
Change from baseline (mg/g)	−2.7 ± 13.8	0.46 ± 5.2	0.365

*Note:* Statistical significance *p*‐value < 0.05.

## 4 Discussion

**FIGURE 4:**
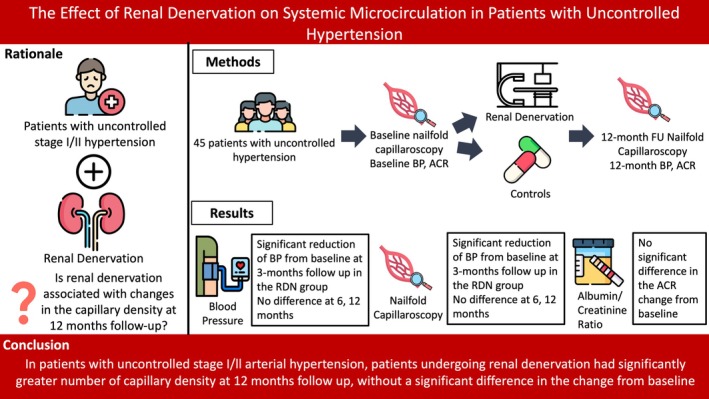
Graphical Abstract. ACR, albumin/creatinine ratio; BP, blood pressure; CD, capillary density; RDN, renal denervation.

This study shows that, in patients with uncontrolled stage I/II hypertension, RDN is associated with more favorable changes in capillary density at 12‐month follow‐up, compared with control patients, as well as with a non‐significant trend toward improvement of albumin excretion levels. Of note, despite numerically higher, the change of capillary density and microalbuminuria between baseline and 12‐month follow‐up was not significantly different (Figure 4). Furthermore, RDN was associated with a significant reduction of BP from baseline, compared with controls, at 3 months, with further reductions at 6 and 12 months not being significantly different between groups. Finally, no procedure‐related safety event was recorded throughout the follow‐up.

Several conflicting data on the effect of RDN in microcirculation have been published. Animal studies in the cerebral microcirculation as well as clinical data on retinal capillaries have indicated a potential benefit of RDN on microvascular function, initiating both structural and functional alterations of the microcirculation [12, 13]. In contrast, evidence on coronary microcirculation and coronary microvascular dysfunction is less positive. A randomized, sham‐controlled study by Engholm et al. [14], evaluating 58 patients with resistant hypertension, found no significant change in transthoracic echocardiography (TTE)‐measured coronary flow reserve (CFR) (0.2 ± 0.2 vs. −0.1 ± 0.2, *p* = 0.57) at 6‐month follow‐up; however, the absence of significant BP reduction in this study could also explain the neutral results. Similarly, Volz et al. [15] also reported no changes in TTE‐CFR (2.7 ± 0.6 vs. 2.7 ± 0.7, *p* = 0.67), baseline and hyperemic mean flow velocity (0.25 ± 0.06 vs. 0.26 ± 0.06, *p* = 0.45 and 0.66 ± 0.15 vs. 0.66 ± 0.13, *p* = 0.94) and resistance index at baseline and hyperemia (0.12 ± 0.37 vs. 0.11 ± 0.22, *p* = 0.16 and 0.27 ± 0.34 vs. 0.31 ± 0.21, *p* = 0.22).

Our study shows a potential beneficial effect of RDN in microcirculation, as we found a significantly greater number of capillaries in those undergoing RDN at 12‐month follow‐up, compared with controls, by approximately 8 capillaries/mm^2^. However, when considering the change of capillary density from baseline, it was not significantly different between groups, despite being numerically greater in the RDN cohort. Animal studies have shown a potential increase in vascular growth factors and nitric oxide bioavailability following RDN [16]; therefore, it is presumable that, besides BP lowering, RDN could initiate both structural and functional changes in microcirculation. Despite this link, a number of reasons may explain the conflicting observations in this cohort. These include the small sample size of 45 patients, which could have limited the power to detect differences from baseline, intra‐observer and inter‐observer variability in the capillaroscopy assessment in the absence of standardized techniques for this test, potential difficulties in assessing the same measurement site as at baseline, and the effect of antihypertensive treatment on capillary density. It is well known that capillary density is related to BP levels and is improved after BP control with antihypertensive treatment [7]. Therefore, the lack of significant change from baseline may be attributed to adequate BP control in all subjects included in this study, limiting the benefit of RDN. Furthermore, despite RDN patients having more advanced disease, as they had significantly higher baseline BP, there was no difference found in baseline capillary density. Thus, more progressed disease does not seem to explain the lack of benefit. On the other hand, considering the aforementioned neutral results of other investigations and given the lack of significant changes in our study, it could be possible that RDN does not have a significant effect in microcirculation overall. Regardless, given the positive notion of the increased number of capillaries at 12 months post‐RDN in this group, further studies are needed in order to better explore the relationship between sympathetic denervation and skin capillary density.

Moreover, our study, despite showing significantly higher ACR levels at both baseline and 12‐month follow‐up in those undergoing RDN, found no significant change, despite the numerical reduction of ACR in the RDN group. The increased baseline ACR levels in the RDN arm are to be expected, as these patients had significantly greater baseline BP levels, thus more advanced disease. Albuminuria has been associated extensively with BP levels [17], as well as sympathetic overactivation [18], and therefore, RDN could be an ideal option for reducing ACR, as it targets both pathophysiologic processes. However, similarly to microcirculation, the effect of denervation in ACR is unclear. A number of studies show reduction in ACR following RDN in patients with resistant hypertension [19, 20, 21], while patients with chronic kidney disease (CKD) may be even more benefited [22]. However, other investigations question the efficacy of RDN in reducing microalbuminuria extent in both patients with resistant hypertension and CKD [13, 23]. Our study comes to confirm the latter data, showing no significant change in the ACR levels 12 months after RDN, despite these patients experiencing a drop in ACR, while controls had an increase. The numerical drop without statistical significance may be attributed to a low number of patients in order to observe statistically significant differences, lack of more extended follow‐up, or variability in antihypertensive medication adherence within the group, which was not tested in the study and therefore might have influenced the results. Nevertheless, future, larger studies are necessary in order to evaluate this relationship more extensively.

Despite not being the primary objective of our analysis, in accordance with randomized clinical trials, our study showcases the safety and efficacy of RDN in reducing in patients with uncontrolled hypertension. This is especially noted at the 3‐month follow‐up, where no alterations had been made in the pharmacotherapy of the patients (based on BP targets), and where RDN was associated with significant reductions in BP in both office as well as 24‐h systolic and diastolic BP. This effect was ameliorated after the 3‐month follow‐up, as no difference was found between the RDN and control group in the BP difference in most of BP measurements and all patients had controlled BP levels. It is possible that similar BP reductions between groups were achieved with increased pharmacotherapy burden in the control group, similarly to the observations of the SPYRAL‐HTN ON‐MED Expansion randomized trial [24]. Regarding safety, no peri‐procedural, post‐procedural or long‐term adverse event related to RDN was noted in this patient cohort, in concordance with the low rates of adverse events reported in other studies.

RDN, as aforementioned, is a guideline‐endorsed intervention in the arsenal of hypertension management. Given its mechanism of action and the systemic effects of renal sympathetic ablation, that is systemic reduction of sympathetic overdrive [25], the focus of research has expanded into examining how RDN affects other pathologies, often coexisting with hypertension. Evidence thus far shows that RDN exerts benefit in heart failure [26], arrhythmia burden [27] and metabolic homeostasis [28], supporting the hypothesis of pleotropic actions. In this context, available evidence from microcirculation studies fail to show a clear benefit of the modality in hypertensive patients, or show a modest effect, as our results indicate. As hypertension and coronary microvascular dysfunction often coexist [29], the results of the IMPRESSION study [30], which will test the effect of RDN on invasive CFR, are largely awaited, as no study has evaluated invasive indices of microcirculation yet. Moreover, identification of markers of adequate response to RDN or evaluation of muscle sympathetic nerve activity (MSNA), in order to correlate the reduction of SNS activation with the microcirculation changes, would provide significant information on the relationship between BP reduction, SNS tone reduction and microcirculation regulation. This, as well as more future studies on the topic, are necessary, enrolling larger sample sizes and spanning a longer‐term follow‐up, in order to identify a trend of denervation toward clinical benefit or no effect at all.

Our study presents some limitations. The small size of the sample and the short follow‐up time do not allow statistically safe comparisons between the microcirculation parameters of the two groups and do not permit to comprehend the eventual long‐term impact of RDN on microcirculation. Furthermore, the absence of measurement of serum markers of SNS activity (plasma norepinephrine) has limited our ability to account for circulatory factors affecting peripheral microcirculation. Similarly, the absence of assessment of SNS activation markers, such as MSNA, which could have acted as a surrogate of RDN efficacy, limits our interpretation. In addition, capillaroscopy, despite the potential clinical applications, has no standardization in technique for quantitating capillary density images, lacks a normal range of capillaries per visual field, and presents intra‐observer and inter‐observer variability. Finally, no testing on the pharmacotherapy burden or adhesion to medication was performed in the patient cohort.

## 5 Conclusion

In patients with uncontrolled stage I/II hypertension, RDN is related to a higher number of capillaries at 12 months compared with controls, without significant difference, however, in the change from baseline. Furthermore, RDN was not associated with significant reductions of ACR, while it was safe and efficient in reducing BP levels. Considering the non‐significant changes from baseline, the small study size, and the limitations of nailfold capillaroscopy, future studies, in both systemic and coronary microcirculation, are needed in order to further determine the role of RDN in microvascular dysfunction.

## 6 Perspectives

Arterial hypertension is extensively linked with microvascular dysfunction and capillary rarefaction, with sympathetic overdrive being one of the common pathogenetic mechanisms. This study shows that, when assessing skin capillaries via videocapillaroscopy at 12 months postrenal denervation, there is a notion of benefit in those undergoing the intervention, compared with controls. Further studies, including larger patient cohorts and long‐term follow‐up, as well as analysis of subgroups based on stage of hypertension, response to RDN and sex, are needed in order to fully explore the effect of renal sympathetic denervation in systemic microcirculation.

## Ethics Statement

Ethics approval was acquired by the responsible Institutional Committee.

## Consent

The authors declare that all patients consented for the participation in the study.

## Conflicts of Interest

The authors declare no conflicts of interest.

## Supporting information


Data S1

## Data Availability

Data are available upon reasonable request from the authors.
