# Supplementary material for: The Effect of Renal Denervation on Capillary Density in Patients With Uncontrolled Hypertension
Source: Microcirculation. 2025 Jun 22;32(5):e70015. doi: 10.1111/micc.70015 (PMC12182759; doi:10.1111/micc.70015)

**Supplementary Material**

**Tables**

Supplementary Table 1: Baseline BP and HR

Supplementary Table 2: BP and HR values at 3,6- and 12-months FU

**Figures**

Supplementary Figure 1: A. Changes in ambulatory BP levels from baseline at 6 months; B. Changes in ambulatory BP levels from baseline at 12 months.

**Tables**

Supplementary Table 1: Baseline BP and HR

|  | Renal Denervation (n=27) | Control  (n=18) | p-value |
| --- | --- | --- | --- |
| Office SBP (mmHg) | 164.3 ± 8.4 | 152.0 ± 10.6 | <0.001 |
| Office DBP (mmHg) | 104.5 ± 7.7 | 96.3 ± 8.0 | 0.001 |
| Office HR (beats/min) | 76.7 ± 10.4 | 74.2 ± 10.8 | 0.438 |
| 24h-SBP (mmHg) | 149.7 ± 4.6 | 144.6 ± 7.8 | 0.008 |
| 24h-DBP (mmHg) | 101.6 ± 13.1 | 92.6 ± 9.0 | 0.014 |
| 24h-SBP (daytime) (mmHg) | 153.2 ± 5.5 | 149.6 ± 8.6 | 0.099 |
| 24h-DBP (daytime) (mmHg) | 103.1 ± 8.9 | 97.3 ± 10.4 | 0.051 |
| 24h-SBP (nighttime) (mmHg) | 143.9 ± 8.0 | 135.2 ± 11.0 | 0.004 |
| 24h- DBP (nighttime) (mmHg) | 94.2 ± 9.7 | 84.6 ± 9.2 | 0.002 |
| 24h-HR (beats/min) | 74.7 ± 8.4 | 74.3 ± 9.6 | 0.869 |
| Abbreviations: SBP: Systolic Blood Pressure; DBP: Diastolic Blood Pressure; HR: Heart Rate | | | |

Supplementary Table 2: BP and HR values at 3,6- and 12-months FU

|  | Renal Denervation (n=27) | Control  (n=18) | p-value |
| --- | --- | --- | --- |
| **Office SBP (mmHg)** | | | |
| 3 months | 143.6 ± 12.7 | 150.9 ± 17.2 | 0.179 |
| 6 months | 137.2 ± 10.2 | 129.6 ± 12.9 | 0.067 |
| 12 months | 133.3 ± 9.5 | 129.3 ± 7.5 | 0.147 |
| **Office DBP (mmHg)** | | | |
| 3 months | 91.8 ± 10.6 | 93.7 ± 14.5 | 0.681 |
| 6 months | 87.4 ± 6.3 | 83.7 ± 8.4 | 0.164 |
| 12 months | 85.0 ± 10.1 | 83.9 ± 6.8 | 0.684 |
| **Office HR (beats/min)** | | | |
| 3 months | 76.9 ± 10.6 | 71.2 ± 13.0 | 0.197 |
| 6 months | 75.1 ± 11.4 | 76.4 ± 13.4 | 0.164 |
| 12 months | 74.0 ± 11.9 | 74.0 ± 10.4 | 0.992 |
| **24h-SBP (mmHg)** | | | |
| 3 months | 137.8 ± 10.5 | 147.5 ± 16.6 | 0.054 |
| 6 months | 132.4 ± 10.9 | 131.1 ± 6.7 | 0.697 |
| 12 months | 129.3 ± 7.8 | 127.7 ± 7.2 | 0.473 |
| **24h-DBP (mmHg)** | | | |
| 3 months | 91.5 ± 10.6 | 98.1 ± 10.4) | 0.127 |
| 6 months | 86.0 ± 8.6 | 83.2 ± 6.4) | 0.297 |
| 12 months | 84.3 ± 9.4 | 81.6 ± 6.5) | 0.297 |
| **24h-SBP (daytime) (mmHg)** | | | |
| 3 months | 141.0 ± 10.7 | 149.9 ± 18.3 | 0.091 |
| 6 months | 136.3 ± 10.2 | 134.2 ± 7.4 | 0.527 |
| 12 months | 131.7 ± 8.8 | 131.4 ± 7.4 | 0.889 |
| **24h-DBP (daytime) (mmHg)** | | | |
| 3 months | 94.8 ± 11.3 | 100.4 ± 11.7 | 0.233 |
| 6 months | 89.8 ± 9.4 | 86.7 ± 6.8 | 0.241 |
| 12 months | 87.3 ± 10.1 | 84.9 ± 5.7 | 0.370 |
| **24h-SBP (nighttime) (mmHg)** | | | |
| 3 months | 133.0 ± 12.5 | 143.9 ± 16.9 | 0.055 |
| 6 months | 126.3 ± 13.8 | 124.9 ± 9.3 | 0.742 |
| 12 months | 125.3 ± 9.0 | 119.3 ± 12.6 | 0.072 |
| **24h- DBP (nighttime) (mmHg)** | | | |
| 3 months | 86.2 ± 11.0 | 95.1 ± 11.5 | 0.54 |
| 6 months | 80.0 ± 8.5 | 76.9 ± 7.7 | 0.280 |
| 12 months | 79.2 ± 9.3 | 74.4 ± 10.7 | 0.120 |
| **24h-HR (beats/min)** | | | |
| 3 months | 75.9 ± 9.3 | 69.9 ± 9.4 | 0.119 |
| 6 months | 74.0 ± 8.6 | 72.5 ± 10.4 | 0.623 |
| 12 months | 74.2 ± 9.3 | 73.7 ± 11.2 | 0.876 |
| Abbreviations: SBP: Systolic Blood Pressure; DBP: Diastolic Blood Pressure; HR: Heart Rate | | | |

**Figures**

Supplementary Figure 1: A. Changes in ambulatory BP levels from baseline at 6 months; B. Changes in ambulatory BP levels from baseline at 12 months. Abbreviations: BP: Blood Pressure; SBP: Systolic Blood Pressure; DBP: Diastolic Blood Pressure; HR: Heart Rate; RDN: Renal Denervation


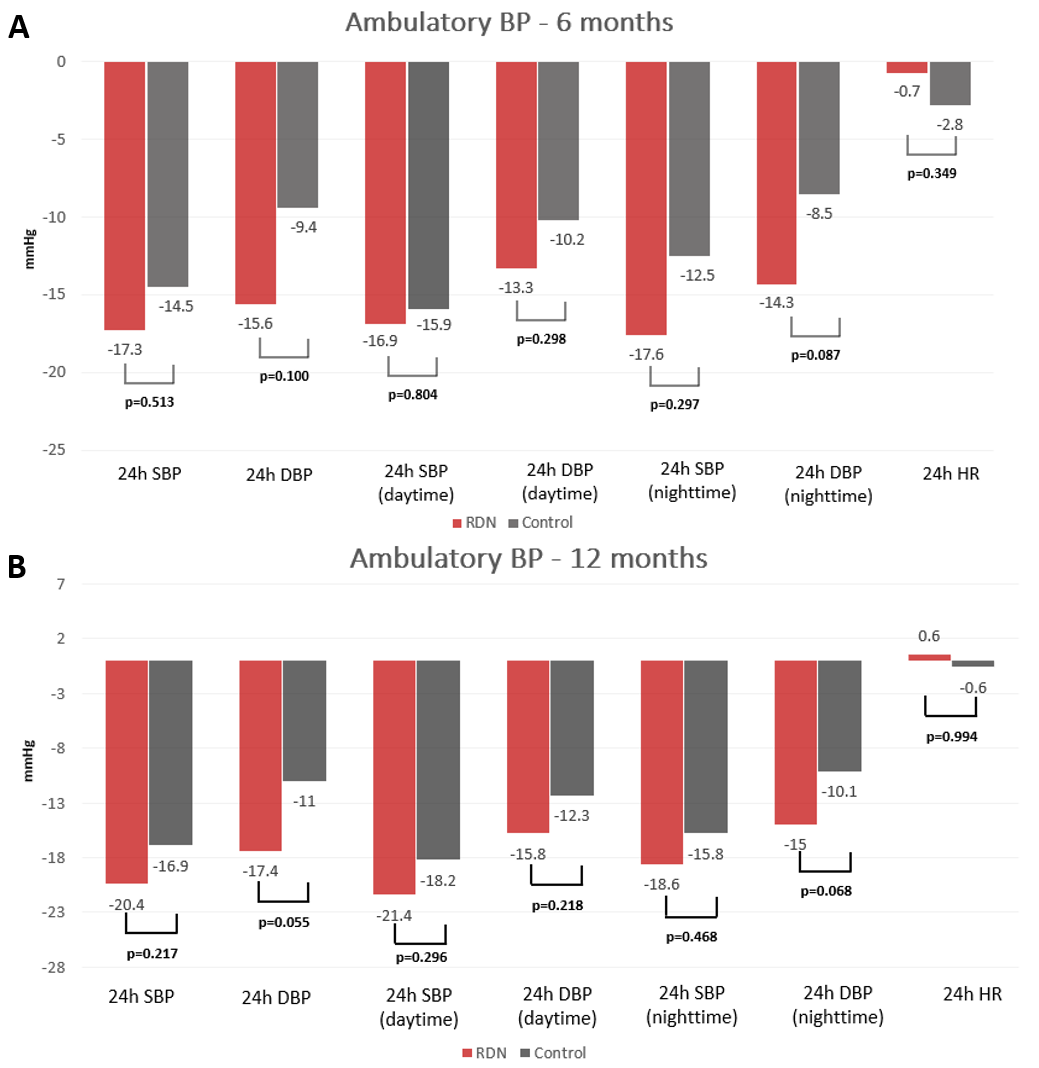

Supplement: Supplementary file 1 — Data S1 [file MICC-32-e70015-s001.docx]
